# Ribonucleoprotein Particles Containing Non-Coding Y RNAs, Ro60, La and Nucleolin Are Not Required for Y RNA Function in DNA Replication

**DOI:** 10.1371/journal.pone.0013673

**Published:** 2010-10-27

**Authors:** Alexander R. Langley, Helen Chambers, Christo P. Christov, Torsten Krude

**Affiliations:** Department of Zoology, University of Cambridge, Cambridge, United Kingdom; University of Minnesota, United States of America

## Abstract

**Background:**

Ro ribonucleoprotein particles (Ro RNPs) consist of a non-coding Y RNA bound by Ro60, La and possibly other proteins. The physiological function of Ro RNPs is controversial as divergent functions have been reported for its different constituents. We have recently shown that Y RNAs are essential for the initiation of mammalian chromosomal DNA replication, whereas Ro RNPs are implicated in RNA stability and RNA quality control. Therefore, we investigate here the functional consequences of RNP formation between Ro60, La and nucleolin proteins with hY RNAs for human chromosomal DNA replication.

**Methodology/Principal Findings:**

We first immunoprecipitated Ro60, La and nucleolin together with associated hY RNAs from HeLa cytosolic cell extract, and analysed the protein and RNA compositions of these precipitated RNPs by Western blotting and quantitative RT-PCR. We found that Y RNAs exist in several RNP complexes. One RNP comprises Ro60, La and hY RNA, and a different RNP comprises nucleolin and hY RNA. In addition about 50% of the Y RNAs in the extract are present outside of these two RNPs. Next, we immunodepleted these RNP complexes from the cytosolic extract and tested the ability of the depleted extracts to reconstitute DNA replication in a human cell-free system. We found that depletion of these RNP complexes from the cytosolic extract does not inhibit DNA replication in vitro. Finally, we tested if an excess of recombinant pure Ro or La protein inhibits Y RNA-dependent DNA replication in this cell-free system. We found that Ro60 and La proteins do not inhibit DNA replication in vitro.

**Conclusions/Significance:**

We conclude that RNPs containing hY RNAs and Ro60, La or nucleolin are not required for the function of hY RNAs in chromosomal DNA replication in a human cell-free system, which can be mediated by Y RNAs outside of these RNPs. These data suggest that Y RNAs can support different cellular functions depending on associated proteins.

## Introduction

Ro ribonucleoprotein particles (Ro RNPs) are soluble complexes in vertebrate cells which are detected by autoimmune antibodies from patients suffering from systemic lupus erythematosis or Sjögren's syndrome [1], [2]. Ro RNPs consist of a structured non-coding RNA termed Y RNA, which is bound by the 60 kDa autoantigen protein Ro60, the 50 kDa autoantigen protein La, and possibly other proteins. The physiological function of Ro RNPs is controversial as divergent functions have been reported for its different constituents.

**Figure 1:**
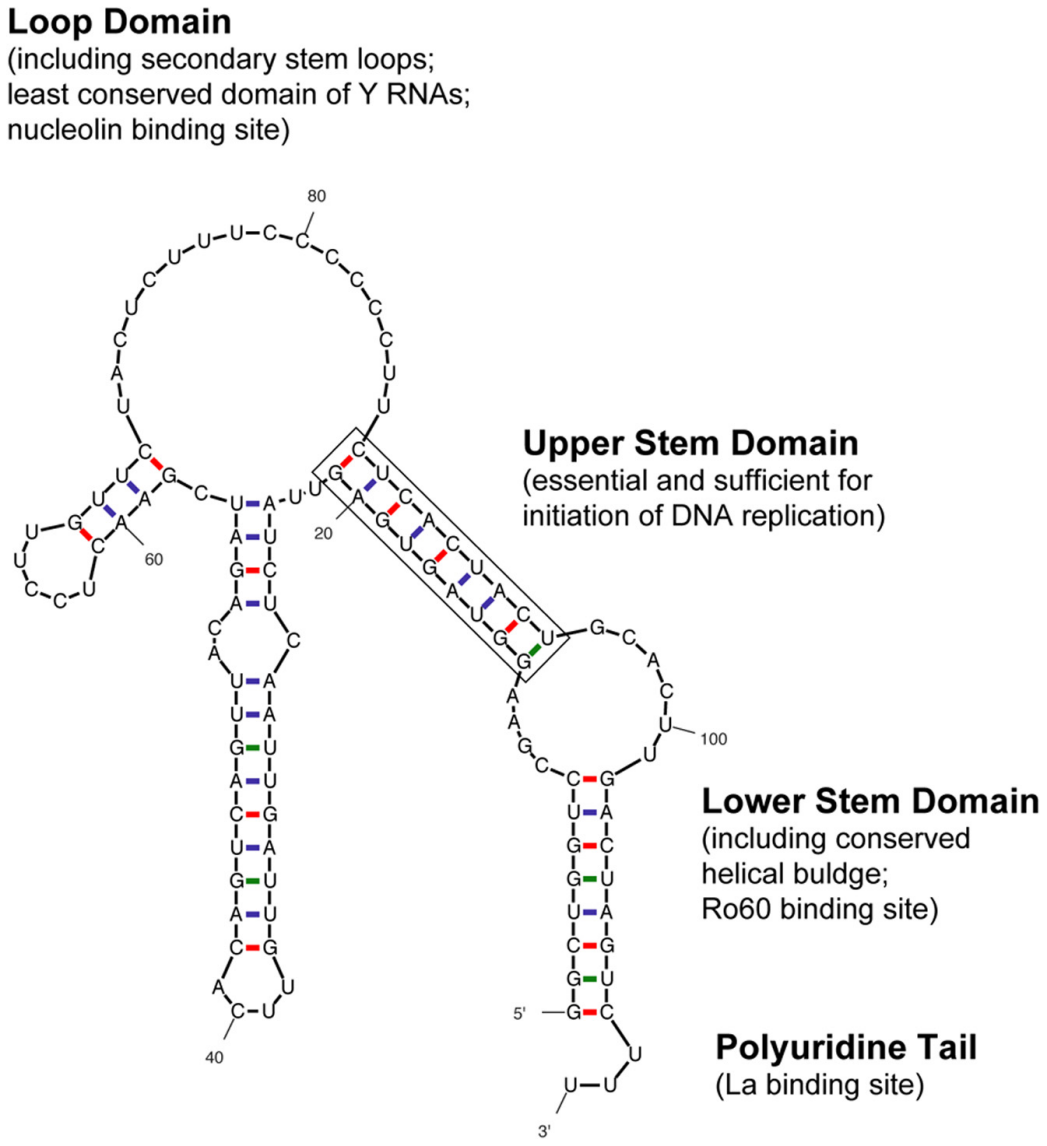
Domain structure of human Y RNA. The most stable secondary structure of hY1 RNA is shown as determined by the Mfold v3.2 RNA algorithm. The four conserved key structural elements, including specific protein binding sites, are indicated.

Y RNAs are RNA polymerase III transcripts of about 100 nucleotides, which fold into characteristic stem-loop structures [3], [4]. They are evolutionarily conserved in vertebrates [5], [6]. Human cells express four Y RNAs (hY1, hY3, hY4 and hY5) [1] and the nucleotide sequence and domain structure of the representative hY1 RNA is shown in Fig. 1. Y RNAs have an essential function for the initiation step of chromosomal DNA replication in mammalian somatic cells [7], [8], [9], [10]. Knockdown of hY RNAs by RNA interference results in the inhibition of DNA replication and the cytostatic arrest of cell proliferation [7], [8], [10]. Additionally, depletion of hY RNAs from human cell extracts specifically inhibits the initiation step of chromosomal DNA replication in a mammalian cell-free system [7], [9]. This inhibition can be overcome by the addition of pure Y RNAs. Recently, we have shown that an evolutionarily conserved double-stranded RNA motif present in the upper stem of vertebrate Y RNAs is essential and sufficient for their function in DNA replication in mammalian cells [10]. These data indicate that Y RNAs are functionally active, but it is unknown if any of the associated Ro RNP proteins play a role in DNA replication.

Ro60 protein binds to a highly conserved site on the lower stem of the Y RNAs [11], [12], [13], which is distinct from the domain required for DNA replication [10]. Ro60 has been proposed to function as a scavenger for mis-folded RNAs in a quality control pathway for non-coding RNAs [13], [14], [15]. Y RNAs have been implicated to play a regulatory role in this process as structural studies have indicated partially overlapping binding sites on Ro60 for misfolded RNAs and Y RNAs [13], [16]. This function of Ro60, however, is not essential as Ro60 knockout mice are viable and, furthermore, derived embryonic stem (ES) cells show no major proliferation defects [17], [18]. Ro60 knockout cells also show reduced levels of Y RNAs, thus implicating Ro60 in RNA stability control [17], [18], [19], [20], possibly by providing protection from exonuclease digestion. Additional studies have demonstrated that Ro60 binding is a prerequisite for the export of Y RNAs from the nucleus to the cytoplasm [21], [22].

La protein is required for the accurate and efficient termination of RNA polymerase III transcription and binds the 3′ polyuridine tail of newly synthesised RNAs in the nucleus [23], [24], [25], [26], [27]. Unlike most RNA polymerase III transcripts, mature Y RNAs retain a 3′ polyuridine tail and thus Y RNAs are believed to maintain a stable association with La [1], [2], [28], [29]. La has also been implicated in many other aspects of non-coding RNA biogenesis, such as protecting newly synthesised RNA against exonuclease digestion [27] and the nuclear retention of RNAs [21]. In addition to Ro60 and La, several other proteins have also been reported to interact with a subset of hY RNAs including the multifunctional protein nucleolin, which associates with the pyrimidine-rich loops of hY1 and hY3 RNA, but not with hY4 or hY5 [15], [30], [31].

Here, we investigate the functional consequences of RNP formation between Ro60, La and nucleolin with hY RNAs for chromosomal DNA replication. Our data show that RNPs containing Y RNAs, Ro60, La or nucleolin are not required for chromosomal DNA replication in a human cell-free system, and that that Ro60 and La proteins do not inhibit this function.

## Results

### hY RNAs are present in several distinct RNP complexes in human cytosolic extract

**Figure 2:**
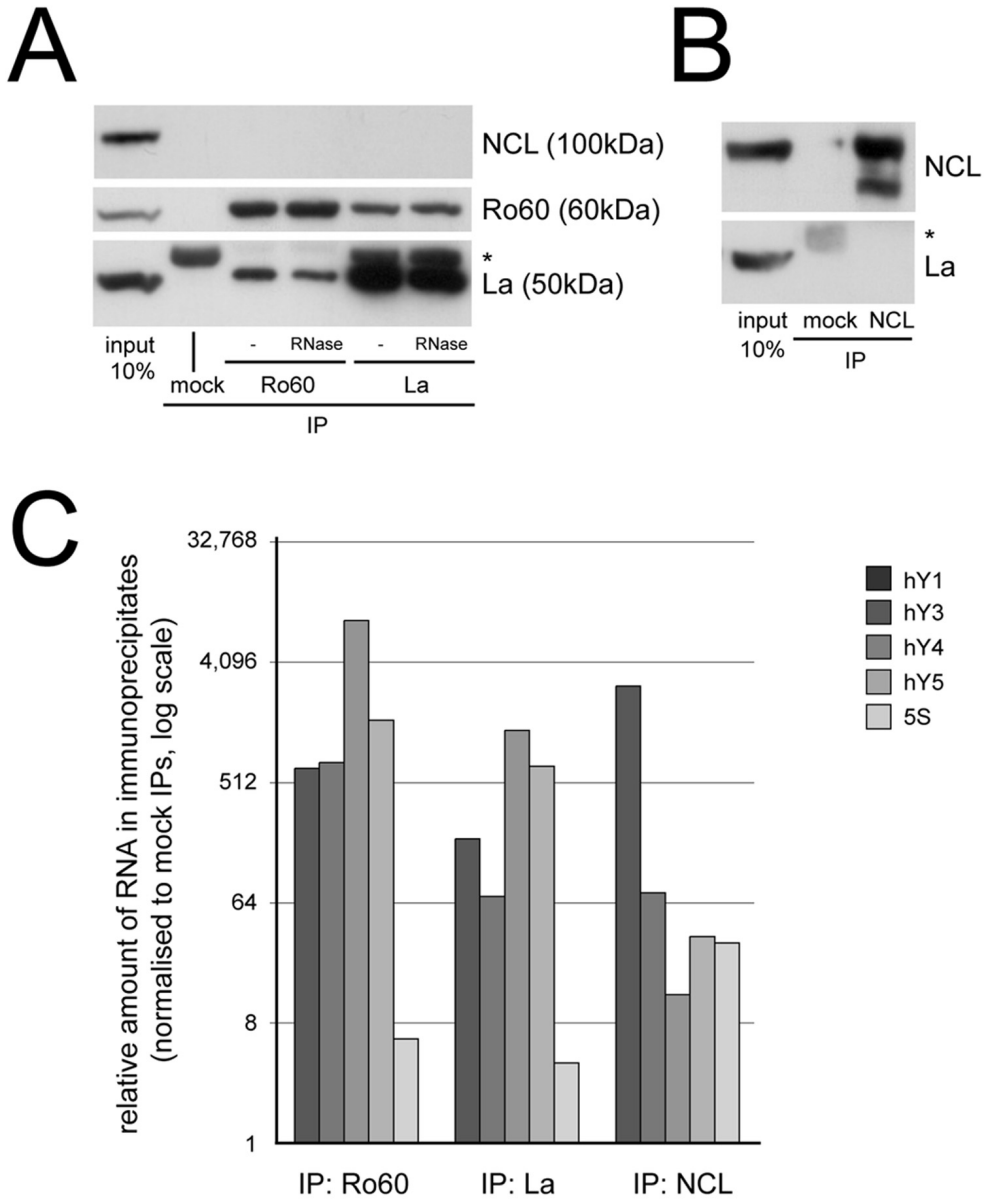
Human Y RNAs are present in several distinct RNP complexes in cytosolic extract. The indicated proteins were immunoprecipitated (IP) from HeLa cytosolic extract and associated proteins and RNAs were analysed by Western blotting and qRT-PCR, respectively. (A) Protein analysis of Ro60 and La IPs. Apparent molecular weights of the precipitated protein bands are shown, the asterisk indicates the IgG heavy chain. As a reference, 10% of the input cell extract was loaded on the gel. Where indicated, extract was treated with RNase A prior to IP (RNase). (B) Protein analysis of nucleolin (NCL) IPs. (C) RNA content analysis of the Ro60, La and nucleolin (NCL) IPs. The relative amounts of all four hY RNAs and 5S rRNA in the indicated immunoprecipitates relative to mock immunoprecipitates were determined by qRT-PCR. Mean values of two independent experiments are shown.

To confirm that Ro60, La and nucleolin stably interact with hY RNAs, we first immunoprecipitated these proteins from HeLa cytosolic cell extract, and then analysed the protein and RNA compositions of these immunoprecipitates (Fig. 2). Anti-Ro60 antibodies precipitated both Ro60 and La, and anti-La antibodies also precipitated both of these proteins (Fig. 2A, bottom panel). Neither anti-Ro60, nor anti-La antibodies co-precipitated nucleolin under these conditions. Conversely, anti-nucleolin antibodies precipitated nucleolin from the extract, but did not co-precipitate La (Fig. 2B). To test whether the co-precipitation of Ro60 and La is mediated by RNA, we treated the extract with RNase A prior to immunoprecipitation. We have previously shown that this treatment reduces the levels of hY RNAs in the extract by three to four orders of magnitude [7]. RNAse treatment did not abrogate the co-precipitation of Ro60 and La (Fig. 2A, bottom panel), suggesting that these proteins either interact directly, or that the close proximity of the binding sites for Ro60 and La on the Y RNA protects the underlying associated RNA fragment from degradation. In any case, these data indicate that Ro60 and La proteins are present in the same complex, while nucleolin is present in a separate complex.

Next, we analysed the RNA content of the immunoprecipitates by quantitative reverse transcription-PCR (qRT-PCR). We determined the relative amounts of the four hY RNAs, and of 5S rRNA as a specificity control (Fig. 2C). All four hY RNAs were enriched more than 500 times in anti-Ro60 immunoprecipitates, compared with mock immunoprecipitates, whereas 5S rRNA was enriched only about 6 fold. In anti-La immunoprecipitates, hY1 and hY3 RNA were enriched 70–200 fold, hY4 and hY5 RNA were both enriched more than 500 times, whereas 5S rRNA was enriched only about 4 fold. In anti-nucleolin immunoprecipitates, hY1 RNA was enriched more than 500 times, while hY3, hY4, hY5 RNA and 5S rRNA were enriched between 15–70 times. We conclude that Ro60 and La stably interact with all four hY RNAs in cytosolic extract, while nucleolin interacts preferentially with hY1 RNA (and to a lesser extent with the other three hY RNAs and 5S rRNA).

Taken together, these data indicate that hY RNAs are present in several distinct types of RNP complexes in the cytosolic HeLa cell extract. First, all four hY RNAs are present in [Ro60/La/hY RNA] RNP complexes. Second, hY RNAs and 5S rRNA are also present in distinct [nucleolin/hY RNA] RNP complexes, which form preferentially with hY1 RNA. Thus, in the next experiments, we investigated the functional requirement of these hY-RNP complexes for chromosomal DNA replication.

### [Ro60/La/hY RNA] and [nucleolin/hY RNA] RNP complexes are not required for the reconstitution of chromosomal DNA replication in vitro

Y RNAs are essential to functionally reconstitute the initiation of DNA replication in a human cell-free system [7]. In this system, isolated late G1 phase template nuclei initiate DNA replication when incubated in cytosolic extracts from proliferating cells [32], [33]. Nucleolytic degradation of hY RNAs present in the extract results in an inhibition of the initiation step of chromosomal DNA replication, which is negated by the re-addition of purified Y RNAs [7], [9]. We therefore used this system to investigate whether [Ro60/La/hY RNA] RNPs and/or [nucleolin/hY RNA] RNPs are required for chromosomal DNA replication.

**Figure 3:**
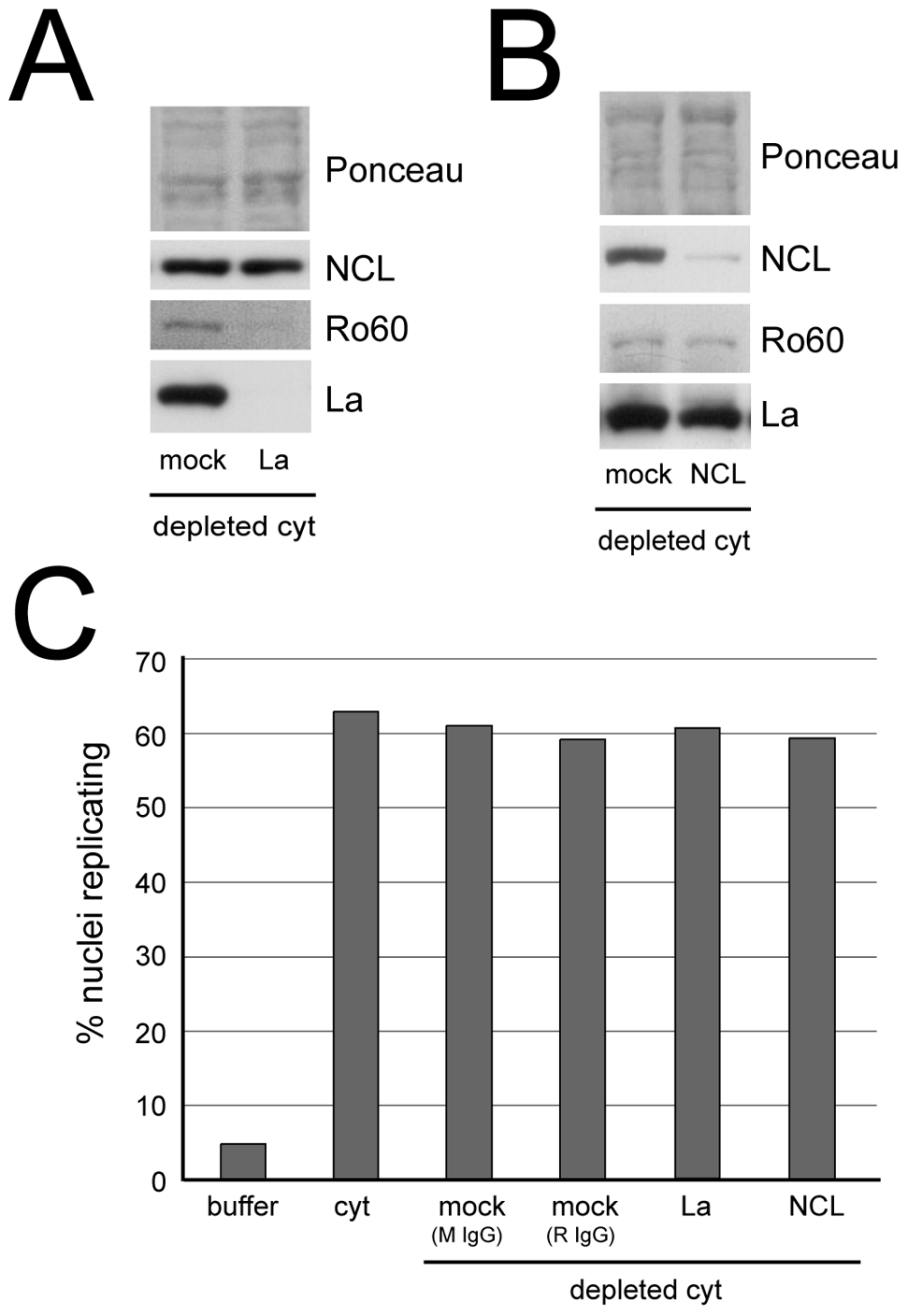
The [Ro60/La/hY RNA] and [nucleolin/hY RNA] RNPs are not required for the reconstitution of chromosomal DNA replication in vitro. Immunodepletion of hY RNPs from cytosolic HeLa cell extracts. (A) Depletion of [Ro60/La/hY RNA] RNPs with La-specific antibodies, and (B) depletion of [nucleolin/hY RNA] RNPs with nucleolin-specific antibodies. Ro60, La and nucleolin (NCL) were analysed in the depleted extracts by Western blot analysis. Ponceau stains are shown as loading controls. For mock depletions, unspecific mouse IgG2a antibodies were used alongside purified mouse monoclonal anti-La antibodies, while pre-immune rabbit serum was used alongside the anti-nucleolin rabbit serum. (C) Functional reconstitution of chromosomal DNA replication in the immunodepleted cytosolic extracts. Late G1 phase template nuclei from mimosine-arrested human EJ30 cells were incubated in the indicated depleted extracts. Replication buffer and non-depleted cytosolic extract (cyt) served as negative and positive controls, respectively. Mock M IgG and R IgG indicate unspecific mouse and rabbit antibodies, as in panels A and B. Proportions of replicating nuclei were determined by immunoflorescence microscopy. Mean values of two independent experiments are shown.

First, we immunodepleted these complexes from the cytosolic extract and then tested the ability of these depleted extracts to reconstitute DNA replication in vitro (Fig. 3). Ro60 and La were both depleted specifically by La-specific antibodies, while the level of nucleolin was unaffected (Fig. 3A). Conversely, nucleolin was specifically depleted by the anti-nucleolin antibodies, while the levels of Ro60 and La were unaffected (Fig. 3B).

We then tested the ability of these depleted extracts to reconstitute the initiation of DNA replication in vitro. When non-depleted cytosolic extract was used as a positive control, 63±4% of nuclei replicated in this extract, compared with 5±4% in buffer alone (Fig. 3C), in agreement with a previous characterisation of this system [32]. Mock depletion of the cytosolic extract with unspecific antibodies did not affect its ability to initiate DNA replication as 61±5% and 59±4% of nuclei replicated in extracts mock-depleted with either mouse or rabbit IgGs, respectively (Fig. 3C). Specific depletion of either [Ro60/La/hY RNA] RNPs or [nucleolin/hY RNA] RNPs resulted in 61±4% and 59±3% of nuclei replicating, respectively. Therefore, depletion of either of these hY RNA-containing RNPs from cytosolic extract has no affect on the initiation of DNA replication in this system, suggesting that they are not required for DNA replication. Furthermore, these results show that the cytosolic protein pools of Ro60, La and nucleolin are also not essential for DNA replication, whether or not these proteins are stably bound to hY RNAs.

**Figure 4:**
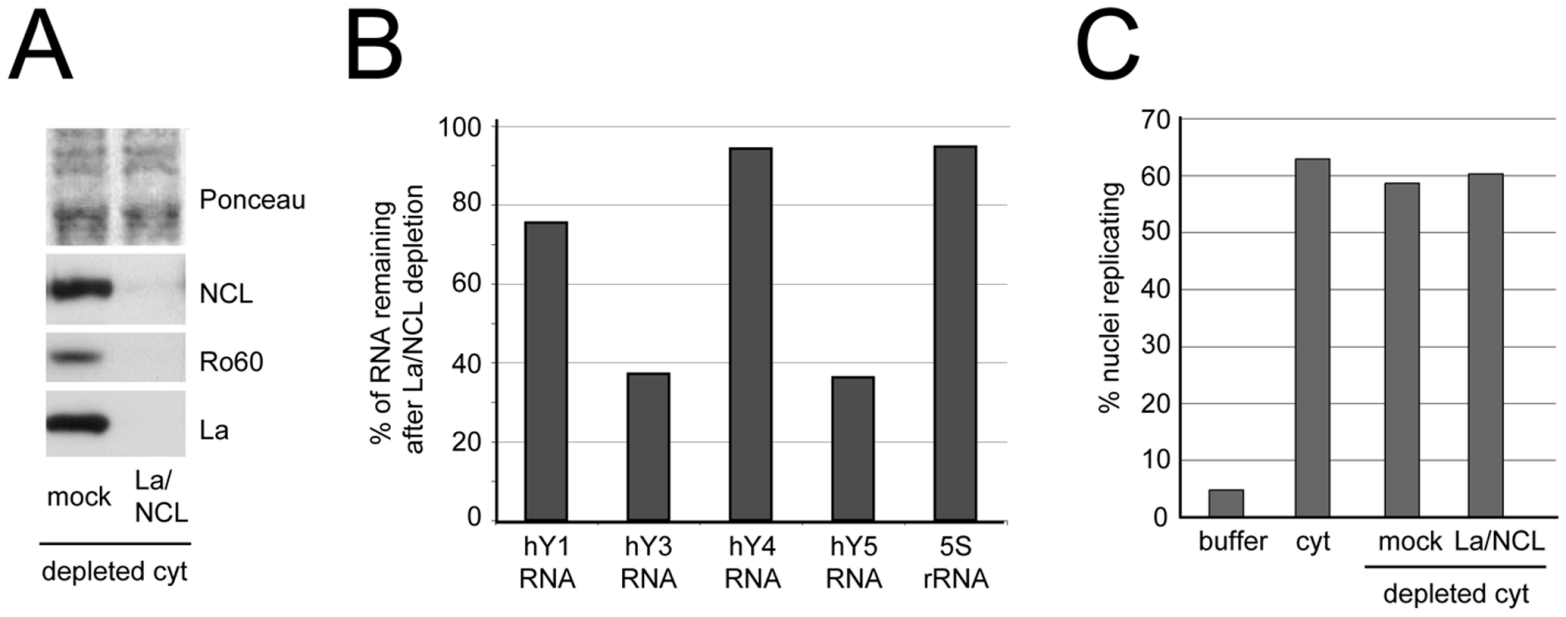
Co-depletion of [Ro60/La/hY RNA] and [nucleolin/hY RNA] RNPs does not inhibit chromosomal DNA replication in vitro. [Ro60/La/hY RNA] and [nucleolin/hY RNA] RNPs were co-depleted with La- and nucleolin-specific antibodies. (A) Protein levels of nucleolin (NCL), Ro60 and La in the depleted extracts were analysed by Western blotting. Mouse IgG2a antibodies and pre-immune rabbit serum were used together for the mock depletion. (B) Analysis of hY RNA and 5S rRNA levels remaining in the depleted extract. Proportions of the indicated RNA amounts remaining in the extract after [Ro60/La/hY RNA] and [nucleolin/hY RNA] RNP co-depletion were determined by qRT-PCR. Data are shown as percentages of the mock depletion, after normalisation to HPRT mRNA. (C) Functional reconstitution of chromosomal DNA replication in the co-depleted extract. Percentages of replicating nuclei were determined as described for Fig. 3C. Mean values of two independent experiments are shown in panels B and C.

We have previously reported that Y RNAs act redundantly with each other in the initiation of chromosomal DNA replication [7], [9], due to an evolutionarily conserved double-stranded RNA motif present in all vertebrate Y RNAs that is essential and sufficient for DNA replication in this system [10]. Therefore, depletion of either [Ro60/La/hY RNA] or [nucleolin/hY RNA] RNPs would still be consistent with redundant roles of either RNP complex in DNA replication. To exclude this possibility, we co-depleted both [Ro60/La/hY RNA] and [nucleolin/hY RNA] RNPs from cytosolic extract (Fig. 4). Ro60, La and nucleolin proteins were all depleted by >99% as determined by Western blot analysis (Fig. 4A). We next determined by qRT-PCR the relative amounts of all four hY RNAs and 5S rRNA remaining in the extract after this co-depletion (Fig. 4B). The relative level of hY1 RNA was reduced to 75% of that seen in the mock-depleted extract, while the relative levels of hY3 and hY5 were reduced to 38%. The levels of hY4 RNA and 5S rRNA, however, were only marginally affected. Taking into account the absolute amounts of these hY RNAs in this cytosolic extract [7], this co-depletion equates to an overall reduction of hY RNAs to about 50% (i.e. from 125±50 nM of hY1-5 RNAs in the undiluted extract [7] down to about 62±25 nM). We conclude that only about half of all hY RNAs in this cytosolic HeLa cell extract are present in RNP complexes with Ro60, La or nucleolin.

We then went on to test the ability of this hY RNP-depleted extract to reconstitute the initiation of DNA replication in vitro (Fig. 4C). Neither mock depletion, nor [Ro60/La/hY RNA]/[nucleolin/hY RNA] RNP co-depletion inhibited the initiation of chromosomal DNA replication in this system as 59±1% and 60±5% of nuclei of nuclei replicated, respectively. We conclude that hY RNAs remaining in the depleted extract, which are not associated with either Ro60, La or nucleolin, are sufficient to reconstitute the initiation of chromosomal DNA replication in vitro.

### Ro60 or La proteins do not inhibit chromosomal DNA replication in vitro

The experiments described above exclude an essential stimulatory function for the major Y RNA-binding proteins Ro60 and La in chromosomal DNA replication in vitro. It nevertheless remains a possibility that these proteins may negatively regulate DNA replication. Ro60 and/or La may sequester Y RNAs and prevent them from functioning in chromosomal DNA replication. To address this possibility, in the last set of experiments, we purified recombinant human Ro60 and La proteins and then tested if they inhibit hY RNA-dependent initiation of chromosomal DNA replication in vitro.

**Figure 5:**
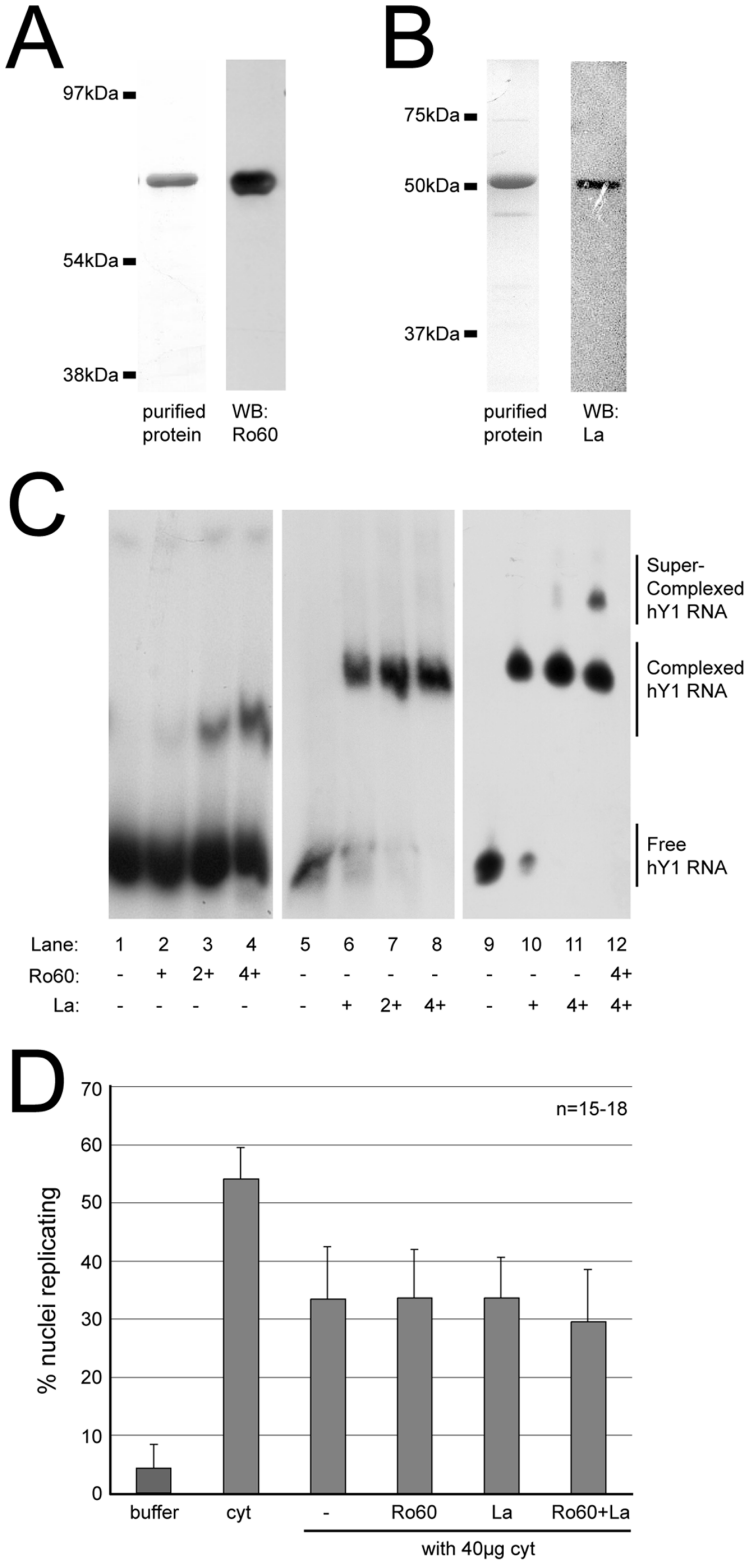
Ro60 and La proteins do not inhibit chromosomal DNA replication in vitro. (A) Protein analysis of purified Ro60 by SDS-PAGE and silver staining is shown on the left panel and Western blot analysis on the right panel. Positions of the molecular weight markers (kDa) are indicated. (B) Protein analysis of purified La by SDS-PAGE and Coomassie staining is shown on the left panel and Western blot analysis on the right panel. (C) Formation of [Ro60/La/hY RNA] RNPs from purified components in vitro. ^32^P-labelled hY1 RNA was incubated with purified Ro60 and/or La at the indicated molar ratios in the presence of a 10-fold molar excess of competitor tRNA. Free RNA was separated from RNPs on native 8% polyacrylamide gels, and radiolabelled RNA was visualised by autoradiography. Positions of free and protein-complexed hY1 RNA are indicated. (D) Functional reconstitution of chromosomal DNA replication in the presence of excess recombinant Ro60 and/or La. Template nuclei were incubated in limiting cytosolic extract (40 µg cyt) with recombinant proteins as indicated. Replication buffer and non-limiting cytosolic extract (cyt) were used as negative and positive controls, respectively. Proportions of replicating nuclei were determined as described for Fig. 3C. Mean values and standard deviations of n = 15–18 independent experiments are shown.

Recombinant human Ro60 was expressed as a poly-histidine tagged protein in E. coli [34], and purified to >99% using Ni-NTA agarose chromatography (Fig. 5A). Recombinant human La was expressed as a chitin binding domain fusion protein in E. coli [34], and purified to >98% homogeneity by affinity chromatography on chitin-agarose, followed by elution of the full-length human La protein after intein-mediated self cleavage (Fig. 5B).

Before using these proteins in replication assays, we first conducted RNA band-shift assays to test their ability to bind purified hY1 RNA (Fig. 5C). Recombinant Ro60 and/or La were incubated with ^32^P-labelled hY1 RNA in the presence of a ten-fold molar excess of unlabelled competitor tRNA to inhibit unspecific binding. Results were analysed on native polyacrylamide gels, followed by autoradiography. Ro60 and La both resulted both in a pronounced upwards shift of the hY1 RNA band in the gel. A four-fold molar excess of Ro60 protein resulted in a shift of about 30–40% of the input hY1 RNA (Fig. 5C, lane 4), whereas equimolar amounts of La were already sufficient to shift >90% of the input hY1 RNA (Fig. 5C, lane 6). This result suggests that La has a higher binding affinity to Y RNAs than Ro60, or that Ro60 may only bind to a fraction of the Y RNAs, such as misfolded ones. Furthermore, addition of Ro60 and La together resulted in a super-shift of the hY1 RNA band above the position obtained with either protein alone (Fig. 5C, compare lane 12 with lanes 4 and 8). These results demonstrate that both recombinant Ro60 and La bind hY1 RNA to form ternary [Ro60/La/hY RNA] RNPs in vitro, indicating that these recombinant proteins are functionally intact.

In the last set of experiments, we investigated the effects of recombinant human Ro60 and La proteins on the initiation of chromosomal DNA replication in vitro. We tested for potential negative effects of these proteins under conditions of limiting cytosolic extract, which allow a more sensitive detection of an inhibition of DNA replication than saturated conditions. The addition of 40 µg of cytosolic extract resulted in only about half the maximal number of nuclei replicating in vitro (33±9%), whereas addition of >100 µg led to the saturation of the system with the maximal number of nuclei replicating (54±5%; Fig. 5D), in agreement with a previous characterisation of the system [32]. We thus titrated recombinant Ro60 or La into the cell free system in the presence of 40 µg of extract. First, we determined by Western blotting that 40 ng and 60 ng of endogenous Ro60 and La were present in the cytosolic extract per 40 µg of total protein, respectively. (This is equivalent to 160 nM and 300 nM of endogenous of Ro60 and La, respectively, whilst all four hY RNAs together are present at 125±50 nM in the extract [7]). Pilot titrations indicated that addition of up to 1 µg of either recombinant Ro60 or La to these conditions did not inhibit DNA replication (data now shown). This result indicates that molar excesses up to 25- or 17-fold of either Ro60 or La protein over total hY RNAs, respectively, do not inhibit the initiation of DNA replication in this system. We finally compared directly the addition of Ro60, La and both of these proteins together to the system (Fig. 5D). Neither the addition of Ro60, La, or of both proteins together at 350 ng had any statistically significant effect on the proportion of nuclei replicating (34±8%, 34±7%, 30±9%, respectively; two-tailed t-tests, unequal variance: P>0.3, n = 15). We conclude from these data that more than 10-fold molar excesses of recombinant Ro60 and/or La proteins over the levels of endogenous hY RNAs do not inhibit chromosomal DNA replication in vitro.

## Discussion

Y RNAs are essential for the initiation of chromosomal DNA replication in mammalian somatic cell nuclei [7], [9]. They were originally discovered as the RNA constituent of soluble, predominantly cytoplasmic, RNPs containing Ro60, La and nucleolin proteins [1], [2], [28], [31], [35]. As it was unknown if these Y RNA binding proteins also play a role in DNA replication, we investigated their function in this process. Our results demonstrate that all four hY RNAs are present in two distinct types of RNP complex in a human cytosolic extract: [Ro60/La/hY RNA] RNPs and [nucleolin/hY RNA] RNPs. We have shown that these RNPs are not required for chromosomal DNA replication in a cell-free system. In fact, immunodepletion of these RNPs removes only about half of the soluble hY RNAs from the extract. As specific degradation of Y RNAs by targeted ribonucleases inhibits DNA replication [7], [9], we conclude that the hY RNA pools, which are not associated with either Ro60, La or nucleolin are sufficient for the initiation of chromosomal DNA replication in vitro. Conversely, we have also demonstrated that an excess of exogenous Ro60 and La does not inhibit DNA replication in vitro. These results indicate that RNP formation between Y RNAs and Ro60, La and nucleolin is not required for Y RNA function in DNA replication, and that these RNPs may thus perform unrelated functions in the cell.

### Distinct hY RNA-containing RNP complexes are present in cytosolic extract

Antibodies against Ro60 or La specifically precipitated Ro60, La and all four hY RNAs, indicating the presence of [Ro60/La/hY RNA] RNP complexes in the HeLa cytosolic extract and confirming previous reports [28], [31], [35], [36]. Degradation of the RNAs present in the extract did not abrogate the co-precipitation of Ro60 and La, also in accordance with a previous report [31]. This is most likely due to the close proximity of the binding sites for Ro60 and La on the Y RNA, thus protecting the associated RNA from degradation.

While anti-nucleolin antibodies precipitated nucleolin and all four hY RNAs, they did not co-precipitate Ro60 or La. Conversely, anti-Ro60 and anti-La antibodies did not co-precipitate nucleolin. Furthermore, our results show that nucleolin is in a complex with 5S rRNA, suggesting that it associates with additional RNAs, as well as with hY RNAs. In contrast to our results, Fouraux and colleagues have reported that anti-nucleolin antibodies precipitated only hY1 and hY3 RNA, but not hY4 or hY5 RNA, and that nucleolin co-precipitates with Ro60 and La [31]. These discrepancies regarding nucleolin-RNPs may be due to the use of different RNA detection methods (qRT-PCR and Northern blotting, respectively) and to the use of different antibodies.

### [Nucleolin/hY RNA] RNP complexes are not required for DNA replication

Nucleolin interacts with pyrimidine-rich stretches in the loop domain of hY RNAs [31], [36]. We have previously shown that deletion of the entire loop domain does not negate the function of Y RNAs in DNA replication [10]. Here, we have shown by immunoprecipitation experiments that the [nucleolin/hY RNA] RNP complexes are not required for DNA replication in vitro, thus independently confirming that the interaction between nucleolin and Y RNAs is not required for the function of Y RNAs in DNA replication.

As result of heat shock or DNA damage, nucleolin rapidly re-localises from the nucleolus to the nucleoplasm and forms a complex with the DNA replication initiation factor RPA [37], [38], [39], [40]. This complex formation sequesters RPA and inhibits the initiation of DNA replication and the G1 to S phase transition. It therefore remains an interesting possibility that nucleolin (with RPA) may also sequester Y RNAs under stress conditions in the intact cell, thus inhibiting the function of Y RNAs in DNA replication under these conditions.

### [Ro60/La/hY RNA] RNP complexes are not required for DNA replication

The main observation of our immunoprecipitation experiments and in vitro DNA replication assays is that RNP formation between hY RNAs, Ro60 and La is not required for Y RNA function in DNA replication. A potential caveat of these in vitro results is the lack of confirmation by an independent experimental approach (e.g. a combined protein knockout in vivo, followed by DNA replication assays). However, our data do directly confirm predictions derived from deletion experiments of the respective protein binding sites from Y RNAs. Ro60 binds to a highly conserved site on the lower stem of Y RNAs [11], [12], [29], while La interacts with the 3′ polyuridine tail [28], [29]. Deletion of these binding sites does not inhibit the function of Y RNAs during chromosomal DNA replication in vitro and in vivo [7], [10]. In contrast, a separate double stranded RNA domain in the upper stem of vertebrate Y RNAs is essential and sufficient for their function in DNA replication [10]. Therefore, either immunodepletion of the entire [Ro60/La/hY RNA] RNP, or deletion of the underlying protein binding sites from the hY RNAs have no effect on Y RNA function in DNA replication. We therefore conclude that Y RNA function in chromosomal DNA replication and Ro RNP function are two separable and independent functions of Y RNAs.

This separation of function allows the reconciliation of controversial reports in the literature. Consistent with our Ro60 immunodepletion data, Wolin and colleagues have reported that Ro60 knockout mice are viable and that derived embryonic stem cells show no major proliferation defects [17], [18]. The levels of mY1 and mY3 RNA in Ro60-knockout cells, however, are reduced, yet no major defects in DNA replication or cell proliferation have been reported [17], [18], [19], [20]. As such, these latter observations could be interpreted as evidence against an essential role for Y RNAs in DNA replication. In contrast, our published data rule out this interpretation, because we have demonstrated that nucleolytic degradation of Y RNAs directly inhibits DNA replication in vitro and in vivo [7], [8], [9], [10]. Like the depletion of Ro RNPs in Ro60 knockout cells, the immunodepletion of Ro RNPs from cell extracts would thus remove a particular Ro RNP-associated subpopulation of Y RNAs with no function in DNA replication, whereas nucleolytic degradation would remove all Y RNAs indiscriminately. Taken together, these data allow the conclusion that those Y RNAs which are not contained in [Ro60/La/Y RNA] or [nucleolin/Y RNA] RNPs constitute a functional relevant pool for DNA replication, while those Y RNAs present in these RNPs may be involved in unrelated processes such as non-coding RNA quality control or RNA stability [14], [17], [19], [20].

Here we have determined by quantitative RT-PCR that about 50% of the hY RNAs in the HeLa cytosolic extract are present in [Ro60/La/hY RNA] or [nucleolin/hY RNA] RNPs and about 50% are therefore outside of these RNPs. This is in contrast to an earlier report [41], showing by Northern blotting that the majority, if not all, cytoplasmic Y RNAs are bound by Ro60 and La. It is possible that these differences arise from different sensitivities of the two RNA detection techniques used. Alternatively, different cells and culture conditions may produce different expression ratios between Y RNAs and Ro60 or La proteins, resulting in different proportions of Y RNAs inside and outside of Ro RNPs. In support of the latter, we have shown that Y RNAs are significantly up-regulated between 5-13-fold in human cancer tissues, compared to normal tissues [8]. This observation suggests that highly proliferative conditions correlate with high Y RNA expression levels, which may result in higher proportions of Y RNAs outside of Ro RNPs.

We do not currently know much about the association of these functionally active Y RNAs with other proteins outside of [Ro60/La/Y RNA] or [nucleolin/Y RNA] RNPs. As it is highly unlikely that the Y RNAs exist as naked RNAs without associated proteins, we therefore speculate that these Y RNAs can associate with proteins involved in the initiation of chromosomal DNA replication. We are currently testing this hypothesis by identifying and characterising novel Y RNA-protein associations in our laboratory and preliminary results show that this is indeed the case. The results of this study will be reported elsewhere.

We found that the addition of more than a 10-fold molar excess of Ro60 and La proteins over the endogenous Y RNA pools did not inhibit the initiation of DNA replication. If one assumes that this extra protein is able to associate with hY RNAs in the extract, one can thus not rule out entirely that Y RNAs may support DNA replication even when associated with Ro60 or La. This scenario is, however, unlikely because La or Ro60 are already present in higher or equimolar amounts to endogenous Y RNAs in the extract, respectively, whilst immunodepletion of more than 99% of total La and Ro60 proteins only removes half of the soluble Y RNAs. It is therefore likely that excess Ro60 and La would be either outcompeted on the Y RNAs in the extract by other Y RNA-binding factors involved in DNA replication, or be bound by additional RNAs with no function in DNA replication, or remain uncomplexed.

An independent precedence for selective targeting of Y RNAs to a cellular pathway independently of Ro RNP formation has recently been reported for retrovirus assembly [19]. Mouse Y RNAs are selectively encapsidated into Moloney murine leukaemia virus (MLV) virions in Ro60-knockout cells as effectively as in wild-type cells, despite significantly reduced intracellular Y RNA levels in the knockouts [19]. By analogy, it is therefore likely that a proportion of cellular Y RNAs is specifically and efficiently recruited for chromosomal DNA replication without involving Ro RNPs. The molecular mechanisms involved in the recruitment of these Y RNAs to intranuclear sites where DNA replication initiates are currently under investigation in our laboratory.

## Materials and Methods

### Immunoprecipitation

Cytosolic extract of asynchronously proliferating human HeLa cells was obtained from Cilbiotech (Mons, Belgium). Extract was diluted in replication buffer (20 mM K-HEPES pH 7.8, 100 mM K-acetate, 1 mM DTT, 1 mM EGTA) and pre-depleted with protein G-agarose or protein A-agarose (both from Roche). (Where applicable, pre-depleted extract was treated with a final concentration of 0.3 mg/ml RNase A (Roche).) Pre-depleted extracts were incubated with the following primary antibodies: anti-60 (Euro-Diagnostica), anti-La (Santa Cruz sc-80655), and anti-nucleolin (a gift from Professor Hans Stahl, University of Homburg/Saar. Germany). Pre-immune rabbit serum and unspecific IgG2a antibodies from murine myeloma (Sigma M5409) were used as negative controls. Antibodies were then precipitated from extracts with protein G-agarose or protein A-agarose (both from Sigma). Protein content of immunoprecipitates was determined via SDS-PAGE followed by Western blot analysis using the same primary antibodies as used for immunoprecipitation. RNA composition was determined by quantitative reverse transcription-PCR (qRT-PCR) following phenol/chloroform extraction and ethanol precipitation.

### Immunodepletion of cytosolic extract

Primary antibodies as used for immunoprecipitation were coupled to protein G-agarose or protein A-agarose beads by incubation in replication buffer. Unspecific IgG2a antibodies from murine myeloma and/or pre-immune serum were used for mock depletions. These antibody-coupled beads were then incubated with cytosolic extract in replication buffer to specifically deplete proteins from the extract. Following three rounds of depletion, protein content of extracts was determined via SDS-PAGE followed by Western blot analysis. RNA composition was determined by qRT-PCR following phenol/chloroform extraction and ethanol precipitation.

### Quantitative reverse transcription-PCR (qRT-PCR)

cDNA libraries were synthesised from RNA preparations of immunoprecipitates or cell extracts using random primers (Promega) with the SuperScript II Reverse Transcriptase kit according to the manufacturer's protocol (Invitrogen). These cDNA libraries were then used as the templates for qRT-PCR on the iCycler iQ platform as described [7]. For RNA-specific cDNA amplification, pairs of primers for hY1, hY3, hY4, hY5, 5S and hypoxanthine ribosyltransferase (HPRT) were used as described [7].

The relative amount (A_R(IP)_) of each hY RNA and 5S rRNA in immunoprecipitates compared with mock immunoprecipitates was calculated from the threshold cycle (C_T_) of each cDNA amplification by using the following equation: A_R(IP)_ = 2^(CT of mock immunoprecipitate – CT of specific immunoprecipitate)^. Identical amounts of starting extract and of protein A or G agarose were used.

In cell extracts, the relative amount (A_R(E)_) of each RNA was first calculated from the threshold cycle (C_T_) of each cDNA amplification by using the following equation: A_R(E)_ = 2^−ΔCT^  =  2^−(CT of test RNA – CT of reference RNA)^. The reference RNA was HPRT mRNA. After immunodepletion of cytosolic extract, proportions of the relative amounts of each RNA were normalised against the mock depleted extract using the following equation: proportion of A_R(E)_ = 2^−(ΔCT of immunodepleted extract – ΔCT of mock depleted extract)^ X 100%.

### Expression and purification recombinant Ro60 and La

For expression of Ro60, the E. coli strain C43 was transformed with the pET28a/his-Ro60 expression vector [34] and grown at 37°C in Lauria broth (LB) to an A_600_ of 0.5. Isoproypylthiogalactoside (ITPG) was added to a final concentration of 0.4 mM, the temperature was lowered to 25°C and growth continued overnight. All purification steps were conducted at 4°C. Bacteria were harvested by centrifugation, washed in PBS, and resuspended in 30 ml protein purification buffer (20 mM KPO_4_, pH 7.8, 300 mM NaCl) with 0.2% Triton X-100 and lysed by sonication. The lysate was clarified by centifugation for 20 minutes at 25,000×g. Imidazole was added to the supernatant to a final concentration of 10 mM, which was then loaded onto Ni-NTA-agarose (Qiagen), pre-equilibrated in purification buffer containing 10 mM imidazole. The column was washed with 40 ml purification buffer containing 150 mM imidazole and Ro60 protein was then eluted with purification buffer containing 600 mM imidazole. The buffer of the eluted protein fractions was exchanged for protein storage buffer (50 mM KPO_4_ pH 7.8, 300 mM KCl, 1 mM EGTA, 0.5 mM DTT, 10% glycerol) using a PD10 column according to the manufacturers protocol (GE Healthcare), before flash freezing in liquid nitrogen and storing at −80°C.

For expression of La, E. coli C43 were transformed with the pTWIN1/La expression vector [34], grown at 37°C in LB to an A_600_ of 0.05. ITPG was added to a final concentration of 1 mM, the temperature was lowered to 26°C and growth continued for 20 h. All purification steps were conducted at 4°C. Cells were washed and lysed in 20 mM Tris-Cl pH 7.0, 500 mM NaCl, 1 mM EDTA, 0.1%Tween 20, 20 µM PMSF by sonication on ice. The lysate was clarified by centifugation for 30 minutes at 20,000×g and loaded onto chitin beads (NEB) and washed in 50 mM Tris-Cl pH 8.8, 1 M NaCl, 1 mM EDTA, 20 µM PMSF. On-column cleavage was induced by addition of elution buffer (50 mM Tris-Cl pH 8.8, 500 mM NaCl, 40 mM DTT, 1 mM EDTA). Eluted protein fractions containing La were pooled, exchanged to protein storage buffer, flash frozen in liquid nitrogen and stored at −80°C.

Concentrations of Ro60 and La proteins in human cytosolic extract were determined by serial dilutions of pure proteins alongside of cytosolic extract, followed by Western blotting.

### RNA band shift assays

Human Y1 RNA was synthesised with SP6 RNA polymerase from recombinant DNA templates in the presence of α-^32^P UTP as described [7]. For band shift assays, 80 ng of radiolabelled hY1 RNA was incubated in band shift buffer (10 mM Tris-HCl pH 7.5 100 mM NaCl, 3 mM MgCl_2_, 1 mM DTT) with recombinant Ro60 and/or recombinant La at molar RNA to protein ratios of 1∶0, 1∶1, 1∶2 or 1∶4, and a molar RNA to protein to protein ratio of 1∶4∶4. Competitor tRNA (Roche) was also added to reactions at 10 fold molar excess of the hY1 RNA to inhibit unspecific binding. Reactions were incubated for 15 minutes at 37°C before being loaded onto native 8% polyacrylamide gels (acrylamide:bisacylamide ratio of 180∶1), which were run at 4°C. Following electrophoresis, gels were dried and radiolabelled hY1 RNA was visualised by autoradiography.

### Cell Culture and DNA replication reactions in vitro

Culture of human EJ30 bladder carcinoma cells, cell synchronisation in late G1 phase by mimosine, and preparation of template nuclei were performed as described [32], [42]. Analysis of DNA replication reactions were performed as described [7], [32], [43], [44].
